# Mid- and Long-Term Effects of Endovascular Surgery and Hybrid Procedures for Complex Aortic Diseases

**DOI:** 10.1155/2019/3247615

**Published:** 2019-04-16

**Authors:** Jiasheng Xu, Yu Zhou, Jingjing Guo, Yu Huang, Yangkai Jiang, Kaili Liao, Weimin Zhou

**Affiliations:** ^1^Department of Vascular Surgery, The Second Affiliated Hospital of Nanchang University, China; ^2^Department of Clinical Laboratory, The Second Affiliated Hospital of Nanchang University, China

## Abstract

**Objective:**

To assess the efficacy and short- and mid-term results of endovascular surgery and hybrid surgical procedures in treatment of complex aortic dissection.

**Methods:**

Clinical data of 90 patients with complex aortic dissection admitted from June 2012 to June 2018 were retrospectively analyzed. Among the patients, 60 cases were male and 30 cases were female, and their ages were ranged from 32 to 79, with an average age of 55 years old; different endovascular techniques and/or hybrid procedures were performed in these patients.

**Results:**

Technical success rate was 100% for the entire group of patients. Type I endoleak occurred in 8 patients immediately after stent-graft placement, which in 2 cases disappeared after a proximal Cuff placement, and the other cases received no special treatment. Follow-up was conducted from 1 month to 72 months, with an average of 36.3 months, and no stent-graft migration or organ ischemia was noted. In the follow-up patients, no type I endoleak occurred but type II endoleak was found in 2 cases, which were cured without treatment; no patient had paraplegia.

**Conclusion:**

Endovascular surgery and hybrid procedures have demonstrable mid- and long-term efficacy in treatment of complex aortic diseases. However, this conclusion still requires multicenter, large-sample studies to further confirm.

## 1. Introduction

Endovascular repair is the first choice for treatment of aortic dissection (AD). Hybrid procedures are the combined application techniques of endovascular repair and various surgical bypass grafts. The treatment of aortic disease by hybrid surgery causes the original complex, high-risk surgery feasible, minimally invasive, and low-risk. In our hospital from June 2012 to June 2018, a total of 283 cases of aortic dissection were treated. 90 cases of complicated aortic dissection were treated with chimney technique, fenestration, and hybrid surgery and achieved good mid- and long-term effects. It is reported below.

## 2. Data and Methods

*Inclusion criteria* were as follows: (1) complying with diagnostic criteria for Stanford type A or stable Stanford type B aortic dissection [1–4] and confirmed by magnetic resonance imaging angiography (MRA) or computed tomography angiography (CTA); (2) aortic dissection grading: grade 1 (typical aortic dissection, with a ruptured avulsed endometrium dividing the aorta into true and false lumens); (3) after examination and approval by the ethics committee of the hospital, the patient and family members are informed and sign the consent form.

*Exclusion criteria* were as follows: (1) mental/psychiatric disorders; (2) serious abnormalities in vital organs such as lung, liver and kidney, or serious diseases of the hematopoietic system and immune system; (3) inability to cooperate or withdraw for various reasons; (4) pregnancy or lactating women;

### 2.1. General Information

There were 90 cases of aortic dissection in this study, 60 males and 30 females, aged 32 to 79, mean 55 years old. The course of hypertension ranged from 8 to 18 months. The general information of patients is shown in Table 1. Among them, 2 cases had a history of coronary stent implantation before operation. All patients were given bed rest, blood pressure control, pain relief, and other symptomatic treatments before surgery. All patients were diagnosed with CTA or MRA. Diagnostic criteria for acute aortic dissection: within 14 days of onset, diagnostic criteria for chronic aortic dissection: 14 days after acute onset or asymptomatic aortic dissection accidentally found during physical examination. Thoracic Endovascular Aortic Repair (TEVAR) was only used for patients with Stanford type B aortic dissection. For patients with Stanford type A aortic dissection, aortic valve junction suspension + ascending aortic replacement was used. For patients with severe aortic tears and involvement of the coronary arteries, Bentall surgery was performed.

### 2.2. Surgical Methods

**Figure 1:**
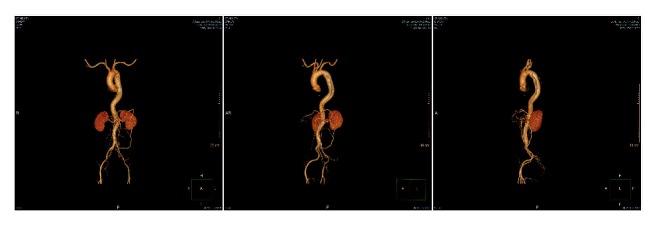
Preoperative CT angiography (CTA) images showing an aortic arch pseudoaneurysm locating between the origin of the left common carotid artery and the left subclavian artery, with a 15-mm tear, 40 mm body and 20 mm sac.

The results of aortic CTA examination (Figure 1) were used for the simulation of three-dimensional reconstruction to determine the treatment plan, the main measurement indicators including the proximal neck diameter (left carotid artery trailing edge level, LSA opening leading edge level, etc.). The proximal diameter of the LSA, the distance between the leading edge of the LSA opening and the left common carotid artery opening, the distance between the first breach leading edge and the LSA opening, and the diameter of the distal neck-neck. According to the measurement results, the proximal and distal diameters of the selected stent, the length of the coating, the diameter and length of the branching stent, and the distance of the backward movement are determined. The center also routinely prepares a proximal Cuff, a distally restricted stent (a large-diameter stent-type stent or a bare stent), and a ball-expanded or self-expanding stent that matches the diameter of the subclavian artery and carotid artery. It is intended to be used when special situations are encountered during surgery. Under normal circumstances, we choose the principle of stent support: the oversize of the dissection case is about 10%; the distance between the LSA and the left carotid artery is less than 6 mm, and the branch support is selected to move backward by 5 mm; if the distance is greater than 6 mm, the backward displacement length is selected. 10 mm bracket; LSA with a diameter greater than 12 mm uses a specially customized 14 mm branch bracket. The operation was performed under DSA surveillance, using endotracheal intubation combined with intravenous anesthesia or continuous epidural anesthesia or local anesthesia. The surgical method is detailed in the author's previous report [1]. If the left side of the vertebral arteries of the patient is of advantage, and it needs to cover the left subclavian artery; then a balloon expansion stent or a coated stent (Fluency, Bard) is implanted inside the left subclavian artery to act as a chimney to ensure the blood supply of left vertebral artery. After stent implantation, superior mesenteric artery stent was implanted if there was residual stenosis in the superior mesenteric artery, and thrombolysis with Unifuse catheter was implanted if there was secondary thrombosis in the superior mesenteric artery. If the access vessel is slender, it is feasible to use the retroperitoneal or transabdominal approach to reconstruct the access vessel and then perform endovascular treatment. For severely distorted iliac artery, the bare stent can be used for support. Iliac artery PTA is feasible when the access vessel is severely stenotic.

## 3. Results

The left subclavian artery (LSA) chimney technique was used in 53 cases, the left common carotid artery chimney technique was used in 2 cases, and the double chimney technique of left common carotid artery and left subclavian artery was used in 1 case. There were 1 case of fenestration using the truncus brachiocephalicus, left common carotid artery and left subclavian artery, 1 case of restrictive bare stent, and 5 cases of vascular artificial blood vessel reconstruction technique. The iliac artery percutaneous transluminal angioplasty (PTA) was performed in 2 cases of aortic dissection patients with severe stenosis of the access vessel. Nine cases were implanted with stent or catheter thrombolysis due to ischemia of superior mesenteric artery (SMA), and 4 cases were treated by hybridization technique. One case of aortic dissection combined with ischemic necrosis of lower extremity arteries was treated with middle and upper thigh amputation in the first stage after TEVAR. Five cases of aortic dissection were narrowed in the distal cavity and implanted with a limiting metal bare stent before implantation of the stent artificial blood vessel; 18 cases of aortic dissection and superior mesenteric ischemia were implanted with the stent. After the operation, the patient was cured by the implantation of the stent in the superior mesenteric artery or thrombolysis with Unifuse catheter. Two cases of Stanford type A aortic dissection were treated with cephalic tract fenestration, left common carotid artery, and left subclavian artery fenestration. Two patients with thoracic and abdominal aortic pseudoaneurysm were treated with superior mesenteric artery sulcus and abdominal cavity occlusion. Eight cases of type I endoleak occurred immediately during operation, and two cases disappeared after releasing a Cuff at the proximal end. Other patients did not receive special treatment. The following six types of endograft were used for endovascular stenting: Hercules (Shanghai Wei Chuang Xinmai)(n=41), Ankura (Shenzhen Xianjian)(n=38), Valiant (Medtronic)(n=17), Relay (Bolton Medical)(n=15), Zenith TX2 (Cook)(n=22), and Fluency (Bard)(n=26). Patient's surgical results and follow-up are shown in Table 2.

**Figure 2:**
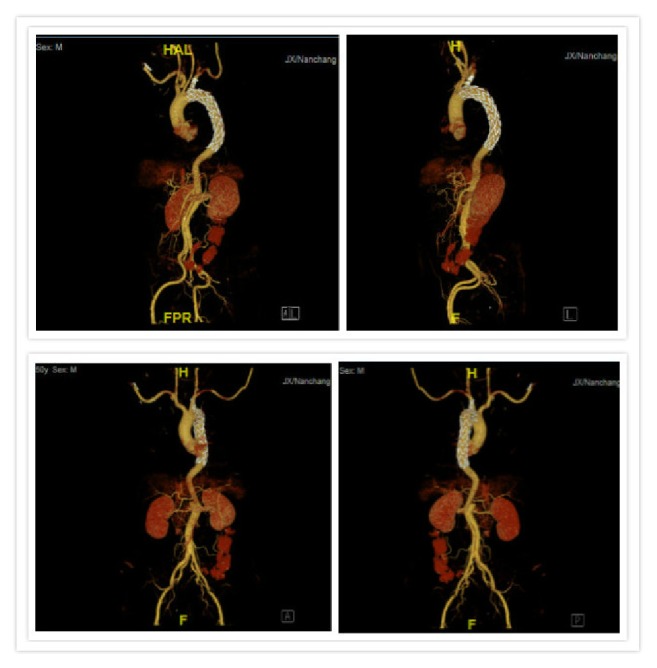
Follow-up CTA on 12 months after operation. Complete thrombus formation of the pseudoaneurysm and patency of the stents.

Follow-up was performed from 3 months to 72 months, with an average of 36.3 months. There were no graft displacement and organ ischemia. There was no type I endoleak in the follow-up patients (Figure 2). Stents of patient treated with chimney technique were unobstructed; 2 cases of type II endoleak occurred and were self-healing with no special treatment. This group has no paraplegia.

## 4. Discussions

### 4.1. Treatment of Insufficient Proximal and Distal Anchoring Regions of Aortic Dissection

In the aortic dissection TEVAR, the proximal part of the stent graft should be at least 1.5~2.0 cm beyond the proximal end of the dissection, which can effectively isolate the true and false lumen of the dissection; try to prevent the opening of the left subclavian artery from being partially or completely covered. If the left vertebral artery is the dominant artery, chimney technique or arterial vascular bypass can be used to remedy [5–11]. Hybrid surgery or chimney techniques can often be used to prolong the anchoring zone when the aortic dissection and abdominal aortic aneurysm are insufficient in the proximal and distal anchoring zones [12–16]. In this group, 26 patients were treated with left subclavian artery, left common carotid chimney technique or left subclavian artery, and left common carotid artery double chimney technique; 1 patient underwent hybrid surgery and left renal artery chimney technique, and 1 patient was recovered by grooving of superior mesenteric artery. There was no endoleak after follow-up and the chimney stent was unobstructed.

### 4.2. Treatment of Poor Vascular Access to

For patients with segmental stenosis of the radial artery, covered stent grafts can be performed after the radial artery PTA. Two patients in this group were cured by this method. For the patients with stenosis of the external iliac artery and the iliac artery, the retroperitoneal or transabdominal approach of the common iliac artery or abdominal aorta artificial vascular reconstruction approach is performed firstly and then endovascular treatment can be performed. In this group, 2 patients with aortic dissection were recovered by TEVAR surgery after common right iliac artery and abdominal aortic artificial vessel approach reconstruction. The follow-up effect was good.

### 4.3. Treatment of Aortic Dissection with Visceral or Lower Limb Ischemia

Patients with aortic dissection often suffer from large pressure in the false lumen due to large rupture and narrow true lumen, which may lead to superior mesenteric artery stenosis or thrombosis, resulting in intestinal ischemia, or lower limb ischemic necrosis due to abdominal aorta or true iliac artery occlusion [17–21]. In most patients, the superior mesenteric artery can restore the blood supply after the open blood supply of the true lumen in the aortic dissection lumen repair, while, in a few patients, the superior mesenteric artery needs to be further treated. Stent implantation is usually feasible for patients with SMA stenosis, with ball-expanded stents as the first choice [22–27]; catheter thrombolysis is feasible for patients with SMA thrombosis. In this group, 9 patients were cured by SMA stenting or catheter thrombolysis in the first or second stage after TEVAR. The author also treated 2 cases of aortic dissection with ischemic necrosis of lower extremity, one case died of ischemic necrosis of both lower limbs due to renal lower abdominal aortic true lumen occlusion caused by type A aortic dissection was not included in the scope of this data, and 1 case of left lower limb necrosis caused by left iliac artery occlusion due to aortic dissection was cured by left lower limb high level amputation after the first stage of TEVAR. In 1 case, aortic dissection led to thrombosis in the false lumen of the right iliac artery and occlusion of the true lumen of the right iliac artery, which resulted in right lower limb ischemia. Although the right lower limb ischemia was slightly improved after TEVAR, there was still right lower limb claudication, which was cured after the right iliac artery stenting in the second stage.

### 4.4. Endovascular Repair and Hybrid Surgery for the Prevention and Treatment of Postoperative Complications of Complex Aortic Diseases

The most common complications of endovascular repair and hybrid surgery are endoleak, stent displacement, aortic injury, distal arterial embolism, spinal cord ischemia, and graft syndrome [26–31]. In this group, type I endoleak occurred in 2 cases, and type I endoleak disappeared after Cuff was applied to the proximal end of the stent graft. Two cases of type II endoleak occurred during follow-up, all of which were appeared after the surgery of EVAR of abdominal aortic aneurysm. One patient was followed up for 3 months and disappeared. One case was followed up for 6 months and disappeared. No complications such as stent displacement and paraplegia occurred.

## 5. Conclusions

In summary, for patients whose left vertebral artery is the dominant artery and the proximal anchoring zone is insufficient, the left subclavian artery or the left common carotid chimney technology is a good solution to the brain blood supply [32–34]. Hybridization technology and window grooving technology can prevent cerebral insufficiency and visceral ischemia [35, 36]. Endovascular repair and hybrid surgery have a good mid- and long-term efficacy in the treatment of complex aortic lesions. However, this conclusion still requires multicenter, large-sample studies to be further confirmed.

## Figures and Tables

**Table 1:** General information of the patients.

Gender	number	age	Stanford A	Stanford B	Acute group	Chronic group	course of hypertension
male	60	32-79	13	41	36	24	10-18 months
female	30	41-72	11	25	18	12	8-16 months
overall	90	55	24	66	54	36	8-18 months

**Table 2:** Patient's surgical results and follow-up.

group	number	Surgical success rate	type I Endoleak	type II Endoleak	Narrow cavity	Superior mesenteric ischemia	stent number	Follow-up time
acute group	54	100%	6	1	3	12	107	3-72 months
choric group	36	100%	2	1	2	6	52	3-72 months
total	90	100%	8	2	5	18	159	36.3 months

## Data Availability

The data used to support the findings of this study are available from the corresponding author upon request.
